# Forgone care and financial burden due to out-of-pocket payments within the German health care system

**DOI:** 10.1186/s13561-014-0036-0

**Published:** 2014-12-20

**Authors:** Patrick Bremer

**Affiliations:** Witten/Herdecke University, Chair for Institutional Economics and Health Policy, Alfred-Herrhausen-Straße 50, 58448 Witten, Germany

**Keywords:** Out-of-pocket payments, Equity, Forgone care, Sample-selection model, Germany

## Abstract

**Background:**

The amount of out-of-pocket (OOP) payments within the German health care system has risen steadily within the last years. OOP payments aim to strengthen patients’ cost awareness and try to restrict the demand on medical necessary treatments. However, besides the intended decline of non-induced health care services there’s a risk that people also forgo necessary treatments because the utilization of health care services depends not only on need-factors but also on the ability to pay for it.

Therefore, this paper aims to analyze the determinants of the total amount of OOP payments, the financial burden caused by OOP payments and the relinquishment of health care services due to OOP payments.

**Data and methods:**

The empirical analysis is based on cross-sectional data of the German subsample (n = 2851) of the Survey of Health, Ageing and Retirement in Europe (SHARE). SHARE is a representative panel study among private households with persons above the age of 50 years and covers a wide range of topics, e.g. health behavior, health status and information about the socio-economic status. The analysis of the independent variables “total amount of OOP payments”, “financial burden due to OOP payments” and “forgone care” is carried out by the means of descriptive as well as multivariate regression methods.

**Results:**

Individuals with low income as well as people suffering from chronic illnesses face a higher financial burden and forgo health care services more frequently at the same time. E.g. the financial burden of people who belong to the lowest income quintile is about eight times higher compared to individuals who belong to the highest quintile. The probability of forgone care for this group is about 5.6 percentage points higher [95% CI: 5.2 – 6.0].

**Conclusion:**

Especially for the group of people with chronic illnesses and low-income earners it cannot be ruled out that they also forgo necessary medical treatments due to the relatively high financial burden they face. Hence, it is required to facilitate the access to necessary care for these groups.

## Background

Between 2002 and 2012 overall health care expenditures in Germany have risen from 228.66 billion € to 300.45 billion €. At the same time, the share of private health care expenditures on total expenditures has risen from 12.1% to 13.5% [1].

The main source of private health care spending within the German health care system are OOP payments. For statutory insured individuals, OOP payments occur for pharmaceuticals, devices, hospitalization and (until January 2013) physician visits. The extent of out-of-pocket expenses for privately insured are regulated in individual insurance contracts, which can contain any type of deductible.

At the micro level it is intended that OOP payments should serve as an instrument to control the use of health care services by reducing the amount of non-necessary or non-effective care (reduction of moral hazard). Therefore, OOP payments aim to reduce public health care spending and to increase the efficiency of medical care by limiting the demand to medically justified services [2,3].

The famous RAND Health Insurance Experiment demonstrated that higher copayment-rates indeed lead to lower levels of medical care use [4]. Generally it was shown that the price elasticity of the demand for health care is not constant but increases as the total amount of out-of-pocket payments rises [5].

However, when analyzing the individual effects of OOP payments on health care use, the determining factor is to a lesser extent the total amount of OOP payments but rather the relation between the amount of OOP payments and the available financial means within a certain period of time – i.e. the financial burden caused by OOP payments.

In this context, OOP payments are known to cause regressive effects [6-8]. That means that individuals with comparatively low income face a higher burden of OOP payments than individuals with higher income.

Previous studies revealed that this burden is not distributed equally among different social subgroups. E.g. in addition to low income groups [9,10], particularly individuals with low education (e.g. [11,12]) women (e.g. [12,13]) and people in poor health [14] bear a higher proportion of OOP payments. At the same time especially the factors “low income” and “chronically ill” are associated with the relinquishment of necessary health care services (e.g. [15,16]), which might result in delayed treatments and adverse health effects [17].

However, the majority of studies which provide evidence for cost induced underuse of medical treatments stem from the US or from low income countries. This means that these findings not necessarily can be translated to Germany because of fundamental differences regarding the organization of the health care system.

E.g., to avoid social disadvantages and an excessive financial burden due to OOP payments in Germany, there exist statutory regulations whereupon people with statutory health insurance only have to pay OOP payments to a certain threshold which is generally 2% and for individuals with chronic illnesses 1% of the household’s gross earnings in one year. When this threshold is reached, patients can apply for an exemption for the rest of the year at their health insurance company [18].

Nevertheless, there is evidence that people who receive exemptions from OOP payments have above-average difficulties in making ends meet before reaching that ceiling [19]. Most studies from Germany which deal with the relationship between OOP payments on the one hand and health care use on the other hand explore the effects of increased OOP payments on doctor visits [20,21]. Only Mielck et al. adopt a broader approach of forgone care by analyzing the association between forgone care (because of costs or unavailability) and household income in five European countries and found that in Germany especially low income is a significant determinant for forgone care [22]. However, relatively little is known about the relationship between the financial burden and forgone care due to OOP payments.

Therefore, the aim of this study is twofold. Firstly, the determinants of the total amount of OOP payments and the financial burden resulting from private health care expenses will be examined and a subsequent analysis focuses on determinants associated with forgone care. Special emphasis is put on factors which influence both the financial burden and the relinquishment of health care services.

The study focuses on people aged 50+ with statutory or private health insurance in Germany. Analyzing the above posed research question on this age group is of particular importance because the demand for health care services and the subsequent amount of OOP payments within this group is above-average due to age-related morbidity [23].

The remainder of this paper is organized as follows: Section two describes the data set and the empirical strategy, in section three the regression results are presented and the final section discusses the results and draws some conclusions.

## Methods

### Data

The empirical analysis is based on survey data of the first wave of the Survey of Health, Ageing and Retirement in Europe (SHARE) which was conducted in 2004. SHARE is a representative panel study among private households with more than 30,000 individuals above the age of 50 years from nineteen European countries and Israel. The survey contains detailed information on a variety of different subjects like health behaviour, health status and socioeconomic characteristics. The data was obtained by computer assisted personal interviews (CAPI), supplemented by a self-completion “drop-off-questionnaire”. The sample in our study was restricted to the German subgroup which comprises a total of 3008 observations. After deleting respondents with missing values, the sample size reduced to n = 2851 observations.

### Variables and empirical strategy

In the following empirical analysis, descriptive as well as multivariate regression techniques will be applied. First of all we will compare the mean values of i) the total amount of OOP payments, ii) the subsequent financial burden and iii) the prevalence of forgone care across five income quintiles. Afterwards, the determinants of the above mentioned variables will be examined using different regression techniques.

The total amount of OOP payments refers to the last twelve months and comprises as sum of expenses for:

- inpatient care (hospital stays)
- outpatient care (doctor visits)
- prescribed drugs
- care in nursing homes, care in day care centres and home care services
- devices

The variable “financial burden” is defined as percentage of individual OOP expenses on available income in the past year. Because of their right-skewed distribution we took the logarithm of the variables “total OOP payments” and “financial burden” for the regression analysis. The variable “forgone care” was created as a dichotomous variable from the question “During the last twelve months, did you forgo any type of (health) care because of the costs you would have to pay?” This question referred to the above mentioned types of care.

The main focus in all regression models lies on the impact of the ability to pay. Hence, variables for the income quintiles are included in all models. The variable “income” is calculated as sum of gross individual income from employment, self-employment, pensions, capital assets income and of transfer and rent payments. In order to get the equivalent disposable income for each individual, the resultant sums were standardized by the household-size square root [24]. Furthermore, we control for socio-demographic characteristics, for indicators of the individual health status and further variables for which economic theory [25] and previous studies (e.g. [26]) suggest that they generally affect the demand for health care services (education and insurance status). Table 1 provides further information about the variables included in the empirical analysis.

**Table 1:** V**ariable description**

**Variables**	**Description**	**Ø (N = 2851)**
**Dependent variables**		
Total OOP payments	total amount of OOP payments in Euro	215.15
Financial burden	Percentage of OOP payment expenses on available income	0.012
Forgone care	Relinquishment of health care services (yes = 1; no = 0)	0.056
**Independent variables**		
*Socio-demographic factors*		
Age		67.32
Age: 50-59	Age between 50–59 years = 1; otherwise = 0	0.35
Age: 60-69	Age between 60–69 years = 1; otherwise = 0	0.39
Age: 70-79	Age between 70–79 years = 1; otherwise = 0	0.19
Age ≥ 80	Age ≥ 80 = 1; otherwise = 0	0.07
Men	Gender: male = 1; otherwise = 0	0.47
*Ability to pay*		
Income	in thousands of Euros	23.7
1. Quintile	if income < 13.176 € = 1; otherwise = 0	0.2
2. Quintile	if income between 13.177 € - 19.651 € = 1; otherwise = 0	0.2
3. Quintile	if income between 19.652 € - 29.423 € =1; otherwise = 0	0.2
4. Quintile	if income between 29.424 € - 50.634 € = 1; otherwise = 0	0.2
5. Quintile	if income > 50.635 € = 1; otherwise = 0	0.2
*Health status*		
1-2 chronic diseases^1^	suffering from 1–2 chronic diseases = 1; otherwise = 0	0.52
≥3 chronic diseases	suffering from ≥3 chronic diseases = 1; otherwise = 0	0.2
1-2 acute health problems^2^	suffering from 1–2 acute health problems = 1; otherwise = 0	0.5
≥ 3 acute health problems	suffering from ≥ 3 acute health problems =1; otherwise = 0	0.83
Depressive symptoms^3^	>3 depressive symptoms = 1; ≤ 3 depressive symptoms = 0	0.27
ADL^4^	≥1 limitation in activities of daily living (ADL) = 1; otherwise = 0	0.09
SAH < good^5^	if self-assessed health is fair, bad or very bad = 1; otherwise = 0	0.44
*Education and health insurance*		
Low education^6^	for ISCED-values between 0–2 = 1; otherwise = 0	0.18
Intermediate education	for ISCED-values between 3–4 = 1; otherwise = 0	0.56
SHI	for persons with statutory health insurance (SHI) = 1; for privately insured = 0	0.89

^1^selectable from a list of 14 chronic diseases ^2^selectable from a list of 12 acute health problems ^3^selectable from the EURO-D scale which consists of 12 depression symptoms. A score of greater than three symptoms is regarded as a cut-off point for a clinically significant depression. ^4^selectable from a list of 6 activities of daily living (ADL) such as showering or dressing ^5^self-assessed health (SAH) measured on a scale from 1 = very good to 5 = very bad ^6^educational levels are measured by the International Standard Classification of Education (ISCED) by the UNESCO. ISCED values range from 0 = pre-primary education to 6 = second stage of tertiary education.

The variables “total OOP payments” and “financial burden” belong to the class of limited dependent variables with a right-skewed distribution, which means they are continuous over the most part of their distribution but have a multitude of observations for a specific value. In this case both variables have values equal to zero in 16.7% of all observations, i.e. these individuals faced no OOP payments and therefore did not experience any financial burden in the past year.

When analyzing these dependent variables the type of distribution has to be taken into account. Therefore, different econometric approaches exist which basically differ from each other regarding the assumption to what extent the binary decision about the utilization of health care (yes or no) depends on the resulting amount of OOP payments.

A possible identification strategy is based on the assumption that the binary decision to seek treatment does not depend on the resulting amount of OOP payments. This kind of decision making process can be modeled by estimating a probit-model for the probability that an individual makes any health care expenses followed by an ordinary least squares (OLS) regression applied only to the subsample of people with nonzero expenditures to estimate the coefficients of the positive levels of OOP payments (two-part model, 2 PM). The constricted application of OLS only to the subgroup of individuals with positive expenditures however raises the risk of sample selection bias and therefore biased estimates [27].

An alternative approach is to estimate a sample-selection model (SSM). The SSM is based on the assumption that the binary decision to demand any kind of care and the choice of how much to spend, is determined by different but correlated factors [28].

In the present analysis, the SSM was estimated by estimating a probit-model for the probability of positive OOP payments as first step, using those results to estimate the inverse Mill’s ratio (IMR) and then running an OLS-model on the positive amounts of OOP payments to correct the selection bias. For reasons of comparability, the paper presents the results of both estimation strategies.

To determine the factors associated with forgone care, we estimated two probit-models. The rationale of model 1 is to get a first insight into the association between forgone care and a person’s income. Therefore, in the first model, we solely controlled for sociodemographic and income variables but didn’t account for health characteristics as we assume that an individual’s health condition might be correlated with its income. In the full model 2 we included all explanatory variables in order to get the isolated effect of the income quintiles.

## Results

### Descriptive statistics

Table 2 gives an overview about the mean values of total OOP payments spent last year (column 1), the subsequent financial burden (column 2) and the prevalence of forgone care (column 3).

**Table 2:** **Out-of-pocket payments, financial burden and forgone care by income quintiles**

**Income**	**∑OOP payments in €**	**OOP payments/income in %**	**Forgone care in %**
1. Quintile	156.25(10.39)	3.1(0.004)	8.9(0.01)
2. Quintile	215.83(25.63)	1.3(0.002)	6.2(0.01)
3. Quintile	203.67(17.4)	0.9(0.001)	5.8(0.01)
4. Quintile	206.02(17.98)	0.5(0.001)	5.4(0.01)
5. Quintile	326.24(57.34)	0.4(0.001)	1.8(0.01)
Ø	215.15(12.18)	1.2(0.001)	5.6(0.04)

From column 1 can be seen, that the average amount of OOP payments spent in the last year is about 215 Euros, whereat this value rises across the quintiles, implying that on average, better off individuals spent a higher amount for health than individuals with lower income. For example, expenditures from individuals in the highest quintile (326.24 €) where more than twice as high compared to expenditures from persons in the lowest quintile (156.25 €).

From column 2 the regressive effect of OOP payments becomes apparent since the percentage share of OOP payments on income decreases as income increases. E.g. the financial burden for the lowest quintile is 3.1% whereas the corresponding values for the fourth and fifth quintile account for 0.5% and 0.4%. Hence, individuals with the lowest income face a financial burden which is about 6 times to 8 times as high.

A positive relationship between financial burden and the relinquishment of health care can be noticed from column 3, where people in the poorest group reported forgone care approximately five times more often than the richest group of individuals.

### Determinants for the total amount of out-of-pocket payments

Table 3 presents the estimation results for “total OOP payments”, whereat column 1 contains the coefficients for the probability of nonzero expenditures within the last twelve months and column 2 (sample-selection model) and column 3 (two-part model) illustrate the results for the absolute amount of OOP payments.

**Table 3:** **Regression results for the total amount of OOP payments**

	**Probit**	**SSM** ^**11**^ **, OLS**	**2 PM** ^**12**^ **, OLS**
**Variables**	**OOP payments >0**	**Total OOP payments**	**Total OOP payments**
Age 50-59^1^	−0.10	−0.20*	−0.23**
	(0.118)	(0.102)	(0.094)
Age 60-69	0.00	−0.06	−0.06
	(0.118)	(0.092)	(0.087)
Age 70-79	0.06	−0.04	−0.02
	(0.130)	(0.097)	(0.092)
Men^2^	−0.14**	0.01	−0.03
	(0.062)	(0.062)	(0.048)
1. Quintile^3^	−0.23**	−0.35***	−0.40***
	(0.104)	(0.112)	(0.092)
2. Quintile	−0.08	−0.26**	−0.28**
	(0.104)	(0.102)	(0.093)
3. Quintile	0.17	−0.28**	−0.23**
	(0.110)	(0.104)	(0.093)
4. Quintile	0.01	−0.13	−0.12
	(0.102)	(0.119)	(0.116)
1-2 chronic diseases^4^	0.61***	0.37*	0.56***
	(0.069)	(0.195)	(0.060)
≥3 chronic diseases	0.67***	0.76***	0.97***
	(0.104)	(0.216)	(0.079)
1-2 acute health problems^5^	0.13*	−0.05	−0.02
	(0.065)	(0.065)	(0.054)
≥3 acute health problems	−0.08	−0.15	−0.17
	(0.096)	(0.072)	(0.067)
Depressive Symptoms^6^	0.17*	0.17**	0.21***
	(0.076)	(0.068)	(0.055)
ADL^7^	0.08	0.60***	0.62***
	(0.132)	(0.090)	(0.084)
SAH < good^8^	0.24**	0.30***	0.36***
	(0.077)	(0.081)	(0.055)
Low education^9^	−0.04	−0.25**	−0.26***
	(0.104)	(0.085)	(0.080)
Intermediate education	0.02	−0.21***	−0.20***
	(0.071)	(0.061)	(0.058)
SHI^10^	0.76***	−0.76**	−0.51***
	(0.084)	(0.262)	(0.088)
Constant	−0.00	5.38***	4.79***
	(0.195)	(0.583)	(0.164)
N	2851	2851	2498
Pseudo R^2^	0.132		
Likelihood Ratio Chi^2^	357.93		
Prob > chi^2^	0.000		
Wald chi^2^(18)		505.21	
Prob > chi^2^		0.000	
Adj. R^2^			0.220
F (18, 2832)			38.02
Prob > F			0.000

Reference categories: ^1^Age > 80 ^2^Women ^3^5. Quintile ^4^no chronic diseases ^5^no acute health problems ^6^no depressive symptoms ^7^no limitations in activities of daily living (ADL) ^8^self-assessed health (SAH) ≥ good ^9^high education ^10^privately insured ^11^Sample-Selection Model ^12^Two-Part Model.

Standard errors in parentheses.

***p < 0.01, **p < 0.05, *p < 0.1.

Again, it becomes clear that in absolute terms, individuals with lower income spent less on OOP payments than high-income earners. This fact becomes evident through negative and significant coefficients for the first three income quintiles. In both models, this negative relationship is most pronounced for the bottom quintile since this group paid 35% (SSM) respectively 40% (2 PM) less on OOP payments compared to the reference group. Furthermore, belonging to the lowest quintile is the only negative predictor regarding the probability of positive expenditures amongst the income-variables (column 1).

Moreover, most variables indicating bad health have a significant and positive impact on total OOP payments as well as on the probability of positive OOP payments.

The major determinant for both the total amount of OOP payments as well as the probability for positive OOP payments is the fact of suffering from multiple chronic diseases. Individuals with at least three chronic diseases spent about 67% (SSM) respectively 97% (2 PM) more on OOP payments compared to their reference group - people without chronic diseases.

Additionally, the variables “low education” and “intermediate education” as well as the fact of being statutory insured have proven to be negative determinants for the total amount of OOP payments.

The likelihood-ratio test between both models has a p-value of 0.78 which indicates that the correlation between both error terms is not significantly different from zero. Therefore, the hypotheses that both parts are independent from each other cannot be rejected.

### Determinants for the financial burden due to out-of-pocket payments

The regressive effect emphasized above also persists in the multivariate analysis (Table 4). This is illustrated by significant and positive estimates for all income-variables and by an increase of the magnitude of all coefficients (column 2 and 3). Thus, although the average sum of OOP payments is lower for low-income earners (Table 3), the resulting burden is the higher, the lower the income is.

**Table 4:** **Regression results for the financial burden due to OOP payments**

	**Probit**	**SSM** ^**11**^ **, OLS**	**2 PM** ^**12**^ **, OLS**
**Variables**	**Burden > 0**	**Total burden**	**Total burden**
Age 50-59^1^	−0.10	−0.22**	−0.21**
	(0.118)	(0.097)	(0.1)
Age 60-69	0.00	−0.04	−0.05
	(0.118)	(0.094)	(0.092)
Age 70-79	0.06	−0.02	−0.02
	(0.130)	(0.103)	(0.103)
Men^2^	−0.14**	−0.03	−0.05
	(0.062)	(0.052)	(0.052)
1. Quintile^3^	−0.23**	1.94***	1.96***
	(0.104)	(0.100)	(0.100)
2. Quintile	−0.08	1.34***	1.39***
	(0.104)	(0.096)	(0.093)
3. Quintile	0.17	1.01***	1.07***
	(0.110)	(0.096)	(0.091)
4. Quintile	0.01	0.65***	0.67***
	(0.102)	(0.121)	(0.078)
1-2 chronic diseases^4^	0.61***	0.56***	0.57***
	(0.069)	(0.065)	(0.064)
≥3 chronic diseases	0.67***	0.92***	0.93***
	(0.104)	(0.084)	(0.083)
1-2 acute health problems^5^	0.13*	−0.02	−0.01
	(0.065)	(0.056)	(0.057)
≥3 acute health problems	−0.08	−0.18**	−0.18**
	(0.096)	(0.071)	(0.074)
Depressive Symptoms^6^	0.17*	0.21***	0.23***
	(0.076)	(0.059)	(0.059)
ADL^7^	0.08	0.65***	0.66***
	(0.132)	(0.091)	(0.095)
SAH < good^8^	0.24**	0.37***	0.39***
	(0.077)	(0.060)	(0.057)
Low education^9^	−0.04	−0.25**	−0.26**
	(0.104)	(0.085)	(0.087)
Intermediate education	0.02	−0.21***	−0.23***
	(0.071)	(0.062)	(0.063)
SHI^10^	0.76***	−0.55***	−0.53***
	(0.084)	(0.092)	(0.093)
Constant	−0.00	−6.49***	−6.52***
	(0.195)	(0.202)	(0.168)
N	2851	2851	2498
Pseudo R^2^	0.130		
Likelihood Ratio Chi^2^	350.64		
Prob > chi^2^	0.000		
Wald chi^2^(18)		1488.57	
Prob > chi^2^		0.000	
Adj. R^2^			0.395
F (18, 2832)			86.72
Prob > F			0.000

Reference categories: ^1^Age > 80 ^2^Women ^3^5. Quintile ^4^no chronic diseases ^5^no acute health problems ^6^no depressive symptoms ^7^no limitations in activities of daily living (ADL) ^8^self-assessed health (SAH) ≥ good ^9^high education ^10^privately insured ^11^Sample-Selection Model ^12^Two-Part Model.

Standard errors in parentheses.

***p < 0.01, **p < 0.05, *p < 0.1.

This regressive effect is most distinctive when comparing quintile 1 and quintile 2, because for the lowest income group (a 194% higher share of OOP payments on income compared to the highest income group) the financial burden is 60 percentage points higher compared to the second lowest income group (a 134% higher share of income compared to the highest income group).

Between quintile 2 and quintile 3 (33 percentage points) as well as between quintile 3 and quintile 4 (36 percentage points) that effect declines considerably (compare column 2).

With the exception of the variable “symptoms”, all health measures which indicate bad health have positive signs. Again, being chronically ill and having limitations in activities of daily living (ADL) show the strongest association with financial burden.

Suffering from at least three chronic diseases causes a 92% higher financial burden (SSM) compared to people with no chronic disease. For individuals with up to two chronic diseases the additional burden accounts for 56% on average.

The coefficients from the sample-selection model and the two-part model rarely differ and the hypothesis that both parts are independent again cannot be rejected (p-value of likelihood-ratio test = 0.75). The probit-coefficients for a non-zero financial burden are identical to the probit-coefficients for non-zero OOP payments in Table 3, which can be explained by the fact that all individuals in that sample have an equivalent household income above zero, so that facing OOP payments automatically leads to a financial burden above zero.

### Determinants for forgone care

Table 5 reports the marginal effects for two different model specifications with forgone care as binary dependent variable. As explained above, special emphasis is put on the relationship between financial burden and forgone care in this analysis. Hence, the highest attention is paid on determinants which have proven to have a significant impact on the financial burden in the previous analysis (i.e. low income and indicators for bad health).

**Table 5:** **Probit regression results for forgone care**

**Variables**	**Model 1 Marginal effects**	**Model 2 Marginal effects**
Age 50-59^1^	−0.022*	−0.002
	(0.012)	(0.013)
Age 60-69	−0.033***	−0.018
	(0.012)	(0.012)
Age 70-79	−0.029**	−0.024**
	(0.011)	(0.01)
Men^2^	−0.004	0.006
	(0.008)	(0.008)
1. Quintile^3^	0.077***	0.056***
	(0.022)	(0.02)
2. Quintile	0.045**	0.033**
	(0.02)	(0.018)
3. Quintile	0.049***	0.042***
	(0.02)	(0.019)
4. Quintile	0.033**	0.031
	(0.02)	(0.017)
1-2 chronic diseases^4^		0.026**
		(0.011)
≥3 chronic diseases		0.035**
		(0.018)
1-2 acute health problems^5^		−0.01
		(0.09)
≥3 acute health problems		−0.014
		(0.013)
Depressive Symptoms^6^		0.045***
		(0.011)
ADL^7^		0.02
		(0.015)
SAH < good^8^		0.014
		(0.009)
Low education^9^		0.009
		(0.013)
Intermediate education		−0.007
		(0.01)
SHI^10^		0.009
		(0.012)
N	2851	2851
Likelihood Ratio Chi^2^	26.69	102.00
Prob > chi^2^	0.001	0.000
Pseudo R^2^	0.021	0.078

Reference categories: ^1^Age > 80 ^2^Women ^3^5. Quintile ^4^no chronic diseases ^5^no acute health problems ^6^no depressive symptoms ^7^no limitations in activities of daily living (ADL) ^8^self-assessed health (SAH) ≥ good ^9^high education ^10^privately insured.

Standard errors in parentheses.

***p < 0.01, **p < 0.05, *p < 0.1.

From model 1 (column 1) can be seen that the probability of forgone care is significantly higher for all four quintiles compared to individuals in the top quintile. This negative relationship between income and forgone care is most pronounced for the lowest quintile. When adding further explanatory variables in model 2 (column 2) the positive impact of quintile 4 looses its significance whereat especially the β-values of the two bottom quintiles reduce considerably but remain significant. This suggests that bad health is associated with low income – a finding which is of importance with respect to forgone health care. The probability of forgone care due to OOP payments is 5.6, 3.3 respectively 4.2 percentage points higher for individuals belonging to the first, second and third income quintile compared to the highest quintile.

Among the health measures, having depressive symptoms and suffering from chronic diseases are the strongest predictors for forgone health care. All other variables don’t show a significant impact.

## Discussion

The main focus of this study was to analyse the determinants of the amount spent on OOP payments within the last twelve months, the resulting financial burden and the probability of forgone care due to OOP payments from people above the age of 50 years in Germany. Special emphasis was put on factors which influence both the financial burden and forgone care.

The empirical analysis of the determinants associated with the amount spent on OOP payments presents a clear picture: people with higher income and people with health problems spent a higher amount on OOP payments compared to their reference group.

The positive relationship between income and the amount of OOP payments is in line with health economic demand theory whereby “health” is classified as normal good with positive income elasticity indicating that the demand for health care rises when income rises [25]. Furthermore it is not surprising that health problems lead to a higher demand for health care and therefore cause higher OOP payments.

Despite the fact that there is a positive relationship between income and the amount of OOP payments, a negative association was revealed between income and the financial burden due to OOP payments. In this context all of the four income variables have turned out to be significant determinants for a comparatively high financial burden.

The positive signs across all income quintiles confirm the regressive effect of OOP payments which was found in several previous studies (e.g. [6,29]), i.e. the economic burden resulting from OOP payments isn’t distributed equally because lower income groups bear a higher proportion of OOP payments than higher income groups. Further, it became apparent that except from having acute health problems, all indicators reflecting bad health are associated with a higher financial burden on average.

Regarding the determinants of forgone care it can be summarized that there is a fundamental connection between financial burden and cost induced relinquishment of health care services: the chronically ill, people with lower income and individuals with depressive symptoms spent a significant higher part of their income on OOP payments and forwent healthcare services more frequently at the same time.

## Conclusion

It has to be pointed out that forgone health care should only be alarming when the health status is affected negatively which most likely tends to be the case when people forgo medically necessary treatments. The relinquishment on medically appropriate health care services can lead to delayed treatments and a further decrease of the health status. Therefore, it can imply higher costs for health care systems in the end. Evidence for cost induced under-supply which led to a deteriorated health status was found in previous studies especially for the elderly (e.g. [30]), the socially weak (e.g. [31]) and for chronically ill individuals (e.g. [32]).

One limitation of this study is that it was not possible to distinguish to which extent people forwent appropriate treatments or unnecessary care, whereat the relinquishment of the latter coincides with the objective of reducing moral hazard.

Another weakness of the study might be its cross-sectional design. This is attributed to the fact that the question concerning forgone care has been posed solely in wave 1 so far. However, it would be desirable to exploit panel observations across time. Particularly the evaluation of the modifications of the statutory regulations regarding OOP payments through the GKV-Modernisierungsgesetz (GMG) in 2004 which led to an increase of OOP payments on the one hand and to an accentuation of the OOP payments exemption limit on the other hand, could provide a deeper insight into the causal effect of OOP payments on the demand for health care services and the relinquishment of health care services. Nevertheless, the results of this study are still of great importance for at least two reasons: First, as mentioned above, OOP payments have risen slightly within the last ten years which might result in a higher burden due to OOP payments on average. Therefor it is reasonable to assume that the issue of forgone care by chronically ill and low-income individuals rather increased than decreased. Second, there are several empirical studies which demonstrate that the prevalence of forgone care did primarily not arise from the relinquishment of doctor visits [20,21]. Hence, it is unlikely that the issue of forgone care by chronically ill and low-income individuals vanished in the course of the abolishment of the consultation fee in 2013.

In general, these results demonstrate the necessity of further research activities to address the question if the existing regulations regarding OOP payments within the German health care system exhibit a barrier to necessary treatments for certain social groups. Particularly for people with chronic illnesses and for low-income earners it cannot be ruled out that they at least partially forgo necessary treatments due to the comparatively high financial burden they face. Hence, it seems to be required to facilitate the access to necessary care for these groups. To achieve this objective, two actions are conceivable: First, the exemption limit could be lowered especially for people with low income and chronic diseases and a second option would be to facilitate the option of a “prospective exemption” e.g. by means of a simple income statement.
